# Ophiopogonin B-induced autophagy in non-small cell lung cancer cells via inhibition of the PI3K/Akt signaling pathway

**DOI:** 10.3892/or.2012.2131

**Published:** 2012-11-09

**Authors:** MEIJUAN CHEN, YUHONG DU, MIN QUI, MINGYAN WANG, KEJUN CHEN, ZHENZHOU HUANG, MIAO JIANG, FEI XIONG, JIANPING CHEN, JING ZHOU, FENGRONG JIANG, LIAN YIN, YUPING TANG, LIHONG YE, ZHEN ZHAN, JIN-AO DUAN, HAI-AN FU, XU ZHANG

**Affiliations:** 1 The Pre-clinical Medicine College, Nanjing University of Chinese Medicine, Nanjing 210046, P.R. China; 2 Department of Pharmacology and Winship Cancer Institute, Emory University School of Medicine, Atlanta, GA 30322, USA; 3 The Pharmacology College, Nanjing University of Chinese Medicine, Nanjing 210046, P.R. China; 4 The First Clinical Medicine College, Nanjing University of Chinese Medicine, Nanjing 210046, P.R. China

**Keywords:** autophagy, natural medicines, non-small cell lung cancer, Ophiopogonin B, PI3K/Akt/mTOR

## Abstract

Ophiopogonin B (OP-B) is a bioactive component of Radix Ophiopogon Japonicus, which is often used in Chinese traditional medicine to treat pulmonary disease. However, whether or not OP-B has any potential antitumor activity has not been reported. Here, we show that the non-small cell lung cancer (NSCLC) cell lines NCI-H157 and NCI-H460 treated with OP-B grow more slowly and accumulate vacuoles in their cytoplasm compared to untreated control cells. Flow cytometric analysis showed that the cells were arrested in G0/G1 phase. Nuclear morphology, Annexin-V/PI staining, and expression of cleaved caspase-3 all confirm that OP-B does not induce apoptosis. Instead, based on results from both transmission electron microscopy (TEM) and the expression of microtubule-associated protein 1 light chain 3-II (LC3-II), we determined that OP-B treatment induced autophagy in both cell lines. Next, we examined the PI3K/Akt/mTOR signaling pathway and found that OP-B inhibited phosphorylation of Akt (Ser473, Thr308) in NCI-H157 cells and also inhibited several key components of the pathway in NCI-H460 cells, such as p-Akt(Ser473, Thr308), p-p70S6K (Thr389). Additionally, insulin-mediated activation of the PI3K/Akt/mTOR pathway provides evidence that activation of this pathway may correlate with induction of autophagy in H460 cells. Therefore, OP-B is a prospective inhibitor of PI3K/Akt and may be used as an alternative compound to treat NSCLC.

## Introduction

Gefitinib and erlotinib, epidermal growth factor receptor tyrosine kinase inhibitors (EGFR-TKIs), have been widely used to treat NSCLC in the clinic. However, their efficacy has been limited by both natural and acquired resistance. Autophagy is known as a type II programmed cell death. It has been found that cell death can occur concomitantly with features of autophagy, and excessive stimulation of autophagy through over-expression of beclin1 suppresses tumorigenesis (1,2).

Autophagy is a multi-step process consisting of initiation, autophagosome formation (nucleation, elongation, and completion), maturation, and degradation (3). Autophagy initiation is complete with the accumulation of the ULK1/2- ATG13-FIP200 complex, which results in development of the isolation membrane, also known as a phagophore. The generation of the complex is regulated by mammalian target of rapamyacin (mTOR), which lies downstream of the class I phosphatidylinositol 3-kinase (PI3K)/Akt pathway. mTOR senses mitogenic stimuli, nutrient conditions, and ATP. The development of the autophagosome is dependent on the class III PI3K complex, which consists of the proteins Vps-34, beclin1, and p150, which all localize to the phagophore and recruit further autophagy-related genes (ATGs) to allow for elongation and completion of the autophagosome. Once the autophagosome is developed, its maturation is complete upon fusion with a lysosome to form an autophagolysosome (4,5).

Constitutive activation of the PI3K/Akt pathway occurs in 90% of NSCLC cell lines, thus, promoting cell survival and resistance to chemotherapy or γ-irradation (6). As a result, inhibition of PI3K/Akt signaling is not only important for induction of autophagic cell death but also essential for finding new treatment for NSCLC.

In our preliminary screening, OP-B was found to be effective in reducing the viability of a panel of human NSCLC cells. Further investigation of its anticancer mechanisms in NCI-H157 and H460 cells showed that OP-B primarily induces autophagy but not apoptosis. Examination of the PI3K/Akt/mTOR signaling pathway showed that OP-B selectively inhibits phosphorylation of Akt both at Ser473 and Thr308 in both of the two cell lines, suggesting that OP-B may be a potential inhibitor of the PI3K/Akt pathway for the treatment of NSCLC.

## Materials and methods

### Materials and reagents

Ophiopogonin B was purchased from Nanjing Ze Lang medical technology company. The compound was initially dissolved in dimethyl sulfoxide (DMSO) (Sigma, USA) as a stock solution before use. For treatment of cells, it was diluted in culture medium to the appropriate concentrations, and the final concentration of DMSO was less than 0.01%. The chemicals used were rapamycin, LY294002 (Cell Signaling Technology), staurosporine, insulin, PI, Alamar blue, and Hoechst 33258 (Sigma). We also used the Alexa Fluor 488 Annexin-V/ Dead cell apoptosis kit (Invitrogen, USA).

### Cell culture

Human non-small cell lung cancer cells lines A549, NCI-H460, NCI-H157, H1299, H1792-2, H1944, NCI-226, H358, H292-G, Hop62, and H522 were obtained from Professor Haian Fu (Emory University School of Medicine, Atlanta, GA, USA). Cells were grown in RPMI-1640 medium (Gibco, USA) supplemented with 10% fetal bovine serum (FBS), 100 U/ml penicillin-streptomycin mixed antibiotics, and cultured under 5% CO_2_ at 37°C.

### In vitro viability assay

Cells were seeded into 384-well plates using a Liquid dispenser in a bio-safety cabinet. Using the liquid handling system, cells were treated with drug the next day for 72 h. The final concentrations used in the assay were 50, 25, 12.5, 6.25, 3.125, 1.56, 0.78 and 0.39 μmol/l in triplicate. A volume of 5 μl/well Alamar blue was transferred into the assay plates for a final concentration of 10%. The plates were exposed to an excitation wavelength of 530 nm, and the emission at 590 nm was recorded to determine whether any of the test compounds fluoresce at the emission wavelength and thus interfere with the assay. Plates were returned to the incubator and the fluorescence was read at 4 h. Data were calculated as the percentage of cell viability by the following formula: the percentage of cell viability = (At/As) ×100%. At and As indicated the absorbance of the test substances and solvent control, respectively. The mean value and standard error for each treatment were determined and the % cell viability relative to control (0.01% DMSO) was calculated. The IC_50_ is defined as the concentration of drug that kills 50% of the total cell population as compared to control cells at the end of the incubation period.

### Cell cycle analysis and apoptosis detection

Cells were treated with or without OP-B for 24 h, and then harvested by centrifugation, washed with ice-cold phosphate-buffered saline (PBS), and fixed in ice-cold 70% ethanol overnight. The cells were then treated with 40 μg/ml RNase at 37°C and then stained with 40 μg/ml PI for 30 min. The percentage of cells in each phase (SubG1, G0/G1, S, and G2/M) was calculated (Becton Dickinson).

For Hoechst 33258 nuclear staining, exponentially growing cells were seeded at a density of 10^5^ cells per well onto heat-sterilized coverslips in 6-well plates. After attachment, cells were treated with or without 10 μmol/l OP-B or 1 μmol/l Staurosporine for 24 h. Subsequently, the cells were fixed (methanol: glacial acetic acid = 3:1) for 10 min, and then dyed (Hoechst 33258, 10 μg/ml) for another 10 min at 37°C. After washing three times with PBS, the cells were observed under a fluorescence microscope (Olympus, Japan).

Apoptosis and dead cells induced by OP-B were assayed using the high content screening (HCS) Kinetic Scan Reader (ThermoFisher Scientific, USA). The principle of the assay is that cells are labeled with a cocktail of fluorescent dyes (including Hoechst 33258 and Alexa Fluor 488 Annexin-V) that indicate the cellular properties of interest, including nuclear structure, cell membrane permeability, and early and late apoptosis. All procedures were performed according to the manufacturer’s instructions. The cells were plated at a density of 8×10^3^ cells/well in each well of a 96-well plate. After culturing for 24 h, cells were incubated with 10 μmol/l OP-B for another 24 h. Thirty minutes before the completion of incubation, a cocktail of fluorescent dyes was added to each well. The cells were then fixed with prewarmed Fixation Solution and washed twice with PBS. Plates were then sealed and ran on an HCS Reader to acquire images. Images were analyzed with HCS software, and the fluorescence intensity of Hoechst 33258 and Annexin-V/PI were calculated.

### Transmission electron microscopy (TEM)

After being exposed to 10 μmol/l OP-B for 48 h, the cells were trypsinized, washed with PBS and fixed in 2.5% glutaraldehyde in 0.1 M phosphate buffer (pH 7.2) overnight at 4°C. The next day, cells were washed three times with 0.1 M phosphate buffer. Thereafter, the cells were fixed in 1% aqueous osmium, dehydrated with increasing concentrations of ethanol (30, 50, 70, 80, 90 and 100%), and embedded in araldite. The ultrathin sections were prepared with a microtome (Leica, Germany) and mounted on copper grids. The samples were stained with 2% aqueous uranyl acetate and lead citrate and observed in a transmission electron microscope (Jeol, Japan).

### Western blot analysis

After treating with different concentrations of OP-B, the cells were lysed in RIPA buffer containing 50 mM Tris/HCl (pH 8.0), 150 mM NaCl, 1% Nonidet-P40, 1% sodium deoxycholate, 0.1% SDS, 0.1 mM DTT, 0.05 mM PMSF, 0.002 mg/ml aprotinin, 0.002 mg/ml leupeptin, and 1 mM NaVO_3_. The protein concentrations of the supernatants were determined by the BCA protein assay. Equal amounts of protein were loaded and separated by 10 or 12% SDS-PAGE and then transferred onto polyvinylidene difluoride membranes. The membranes were incubated overnight with appropriate primary antibodies against p-PDK1(Ser241), p-Akt (Ser473), Akt, p-p70S6kinase (Thr389), p70S6kinase, p-4E-BP1 (Thr37/46), 4E-BP1, LC3A/B, caspase-3, Bcl-2, cleaved-caspase-3, or β-actin overnight at 4°C, and then with HRP-conjugated secondary antibodies (anti-rabbit or mouse immunoglobulin G) for an additional 1 h at room temperature. Immunoreactivity was detected by enhanced chemiluminescence (ECL, Bio-Rad). β-actin was used as a loading control. Immunoblot experiments were performed at least three times. Quantitative analysis was performed by Image Lab™ software.

### Statistical analysis

Unless otherwise stated, data were expressed as the mean ± SD, and analyzed by Student’s t test. P-value <0.05 was considered statistically significant.

## Results

### Effect of OP-B on proliferation of NSCLC cells

**Figure 1:**
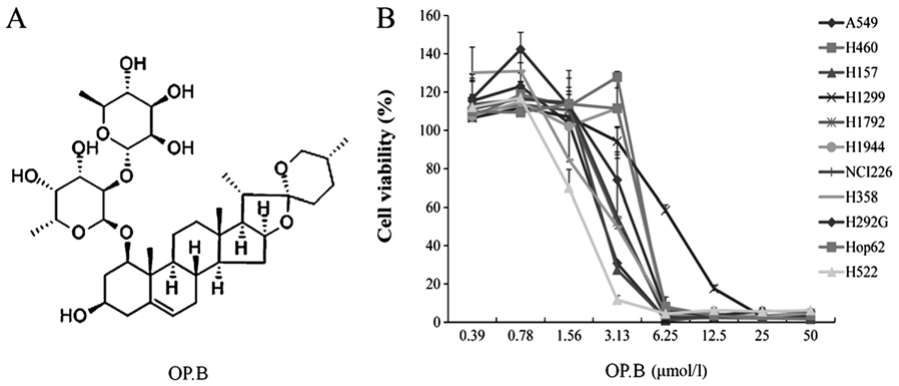
Dose-dependent effect of OP-B on cell viability in NSCLC cell lines. (A) Structural formula of OP-B. (B) Cell viability of eleven NSCLC cell lines 3 days after exposure to increasing doses of OP-B, actual cell numbers were counted with Alamar blue dye. Results shown are the means of three independent experiments; bars, SD.

NSCLC, including squamous carcinoma, adenocarcinoma, and large cell carcinoma, represents ~80–87% of all lung cancer cases (7). To determine whether OP-B (structure shown in Fig. 1A) has any therapeutic effect on NSCLC cells, we performed a cell viability assay using eleven human NSCLC cell lines. After 72 h of treatment, OP-B significantly decreased cell viability of all cell lines tested in a dose-dependent manner, and the IC_50_ was <4 μmol/l (~3.87 μmol/l) (Fig. 1B).

### Effect of OP-B on cell morphology, cell cycle, and apoptosis in NCI-H157 and H460 cells

**Figure 2:**
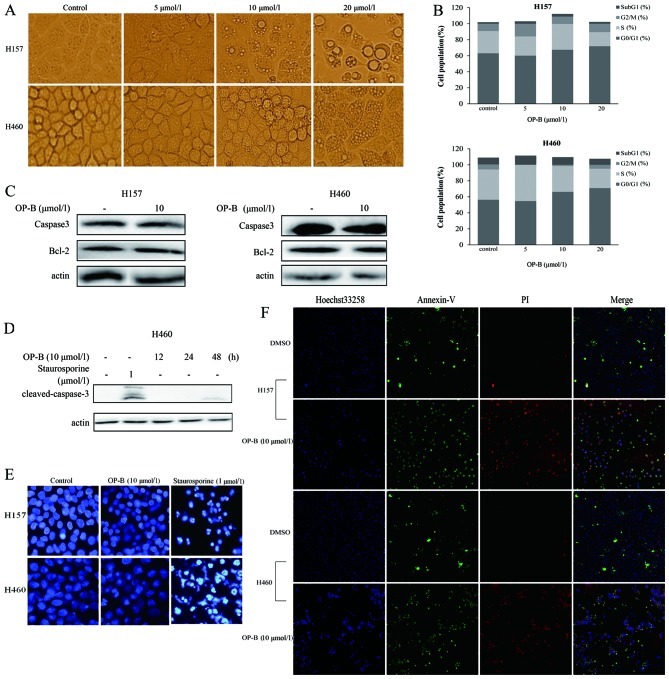
Effects of OP-B on NCI-H157 and H460 cell cycle and apoptosis. (A and B) NCI-H157 and H460 cells incubated with varying concentrations of OP-B for 24 h: (A) cell images were taken by phase-contrast microscope; (B) cell number percentage in each phase (sub-G1, G0/G1, S, and G2/M) was calculated and expressed. (C) NCI-H157 and H460 cells treated with 10 μmol/l OP-B for 24 h were analyzed by immunoblotting with antibodies against caspase-3, Bcl-2 and actin. (D) NCI-H460 cells treated with 10 μmol/l OP-B for 12, 24, 48 h or 1 μmol/l Staurosporine for 12 h were analyzed by immunoblotting with antibodies against cleaved caspase-3 and actin. (E) NCI-H157 and H460 cells treated with 10 μmol/l OP-B or 1 μmol/l Staurosporine for 24 h were stained by Hoechst 33258 and observed by fluorescence microscopy. (F) Cells were incubated with OP-B for 24 h and then fixed and stained with Hoechst 33258 and Alexa Fluor 488 Annexin-V/Dead cell apoptosis kit. Images of cells were taken by the high content screening (HCS) KineticScan Reader (x200).

NCI-H157 and H460 cells represent the main subtypes of NSCLC and are derived from squamous cell carcinoma and large cell carcinoma, respectively. In our experiment, we found that these two cell lines were more sensitive to OP-B (IC_50_ of H157 and H460 was 2.86 and 4.61 μmol/l, respectively) than all other NSCLC cell lines tested. Therefore, we chose these lines to further investigate the pharmacological effect of OP-B. Following 10 μmol/l OP-B treatment for 24 h, many vacuoles appeared in both cell lines but especially in H157 cells (Fig. 2A). When the concentration of OP-B was increased to 20 μmol/l, the vacuoles became even larger and occupied almost all of the space outside the nucleus in NCI-H157 cells.

Flow cytometric analysis of cells stained with propidium iodide showed a mild increase in the cell population in G0–G1 phase after the cells were treated with different concentrations of OP-B for 24 h in medium containing 10% FBS (Fig. 2B). To further investigate whether the cell cycle arrest was associated with apoptosis, we measured levels of caspase-3 and Bcl-2. Treatment with staurosporine served as a positive control for cleaved caspase-3 staining. The results showed that there was no change compared to the negative control (Fig. 2C), and cleaved caspase-3 was only slightly increased in NCI-H460 cells following 48 h of treatment with OP-B (Fig. 2D); however, no change was detected in NCI-H157 cells (data not shown). Nuclear staining with Hoechst 33258 also showed no characteristics of apoptosis, such as cell shrinkage, nuclear condensation, and fragmentation (Fig. 2E). Furthermore, the cells labeled with a cocktail of fluorescent dyes (including Hoechst 33258 and Alexa Fluor 488 Annexin-V/Dead cell apoptosis kit) and scanned with high content screening (HCS) Kinetic Scan Reader (ThermoFisher Scientific) (Fig. 2F) also suggested that OP-B did not induce apoptosis or necrosis in either of the two cell lines.

### OP-B induces autophagy in NCI-H157 and H460 cells

**Figure 3:**
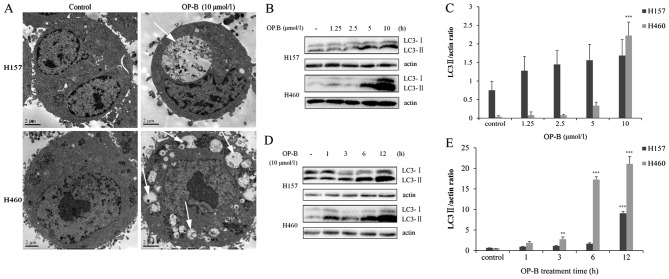
OP.B dose- and time-dependently induces the formation of LC3-II, and induced autophagic vacuole in NCI-H157 and H460 cells. (A) Transmission electron microscopic examination of NCI-H157 and H460 cells treated with 10 μmol/l OP-B for 48 h. Numerous autophagical vacuoles with typical double-layer membrane containing organelle remnants are highlighted by arrows. (B and D) NCI-H157 and H460 cells treated with various concentrations of OP-B for 24 h or 10 μmol/l OP-B for 0, 1, 3, 6, 12 h were analyzed by immunoblotting with antibodies against LC3 and actin. (C and E) Densitometry analysis of LC3- II levels relative to actin was performed using three independent experiments. Error bars, SD; ^**^p<0.01; ^***^p<0.001.

Results from transmission electron microscopy (TEM) showed that the cytoplasmic vaculoes had double-layered membranes and that many of them contained cytoplasmic organelles or myelin figures. Furthermore, the vacuoles increased in size and number and fused into larger vacuoles, while the nucleus remained intact (Fig. 3A). Detection of LC3 by immunoblotting showed that OP-B treatment increased the conversion of LC3- I to LC3-II in a dose- and time-dependent manner (Fig. 3B-E). Thus, we speculated that treatment with OP-B induced autophagy of NCI-H157 and H460 cells.

### Effects of OP-B on PI3K/Akt/mTOR/p70S6K signaling pathway and induction of autophagy in NCI-H157 and H460 cells

The PI3K/Akt/mTOR/p70S6K signaling pathway, which is often associated with tumorigenesis and activated in numerous tumors, is well-known to regulate autophagy (8–10). Thus, the pathway was examined in relation to OP-B-induced autophagy in NCI-H157 and H460 cells.

**Figure 4:**
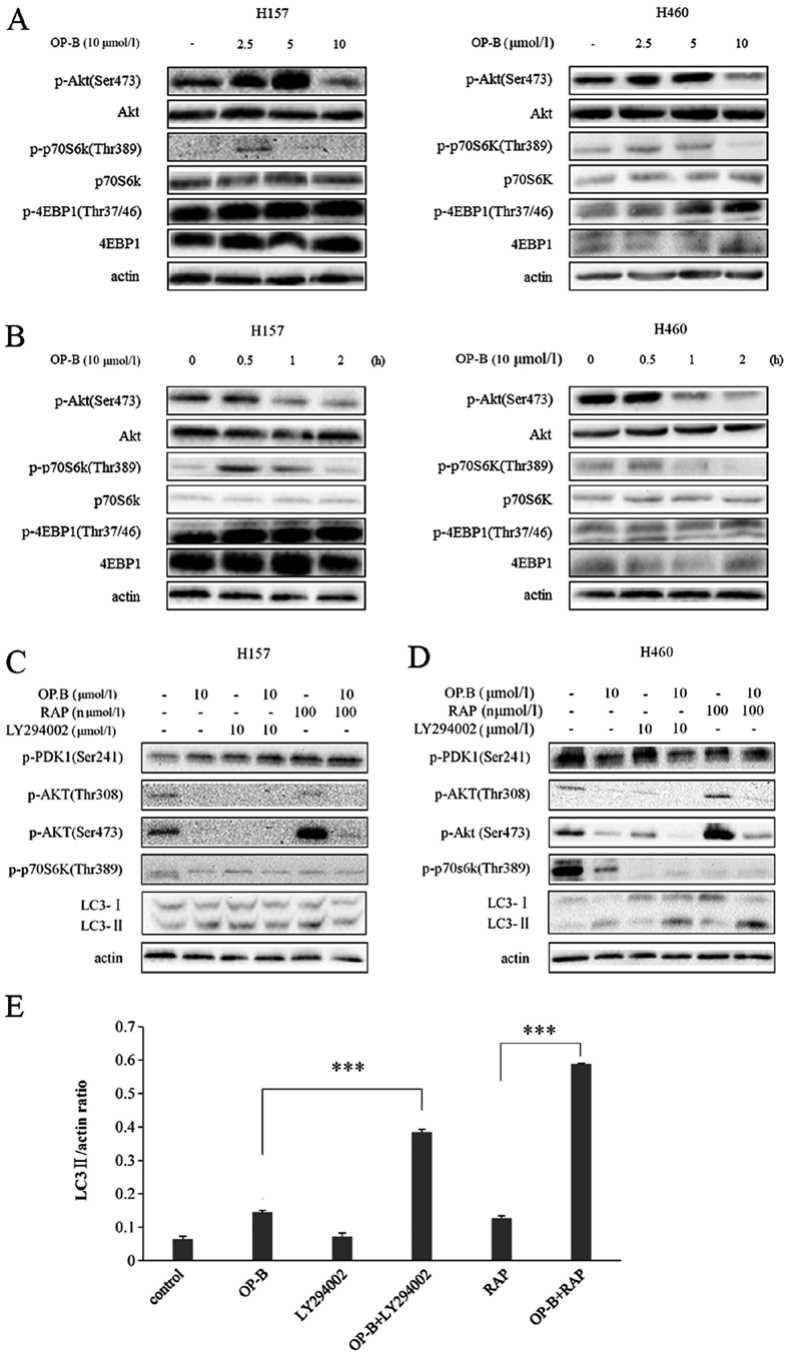
Effect of OP-B on PI3K/Akt/mTOR/ p70s6k signaling pathway in NCI-H157 and H460 cells. (A and B) The NCI-H157 and H460 cells treated with 0, 2.5, 5, 10 μmol/l OP-B for 1.5 h or 10 μmol/l of OP-B for 0, 0.5, 1 or 2 h were analyzed by immunoblotting with antibodies against p-Akt (Ser473), p-p70S6K (Thr389), p-4EBP1 (Thr37/46), Akt, p70S6K, 4EBP1, and actin. (C and D) The NCI-H157 and H460 treated with 10 μmol/l OP-B, 10 μmol/l LY294002 or 10 μmol/l Rapmycin for 4 h were analyzed by immunoblotting with antibodies against p-PDK1 (Ser241), p-Akt (Thr308), p-Akt (Ser473), p-p70S6K (Thr389), LC3, and actin. (E) Densitometry analysis of LC3-II levels relative to actin in H460 cells was performed using three independent experiments. Error bars, SD; ^**^p<0.01; ^***^p<0.001.

As shown in Fig. 4A and B, when cells were treated with at least 10 μmol/l OP-B for at least 1h, p-Akt (Ser473) was significantly inhibited in both NCI-H157 and H460 cells, but p-p70S6K (Thr389) was inhibited only in H460 cells under the same conditions. Under all treatments, p- 4EBP1 (Thr37/46) was not affected.

LY294002 is a well-characterized inhibitor of PI3K, and rapamycin is an inhibitor of mTORC1. Next, we tested the effect of OP-B on the PI3K/Akt/mTOR/p70S6K pathway in both H157 and H460 cells. In H157 cells, similarly to LY294002, OP-B inhibited phosphorylation of Akt both at Ser473 and Thr308, and it weakened the feedback activation of rapamycin on p-Akt at Ser473. In contrast, p-PDK1 (Ser241), p-p70S6K (Thr389), and LC3, were not affected by any of the above treatments (Fig. 4C).

Similarly, in H460 cells, OP-B showed enhanced inhibition of p-Akt (Ser473 and Thr308) after co-treatment with LY294002. Additionally, it weakened the feedback activation of rapamycin on the two sites. Unlike in H157 cells, the phosphorylation of p70S6K (Thr389) was inhibited by treatment with OP-B, LY294002, or rapamycin. Conversion of LC3 I to LC3-II was induced by OP-B, inhibited by LY294002, and enhanced by co-treatment with OP-B and LY294002. In fact, co-treatment with OP-B and rapamycin had an even more significant effect than single treatment with OP-B or rapamycin alone (Fig. 4D and E).

The above results show that in NCI-H157 and H460 cell lines, OP-B has similar pharmacological effects on the inhibition of p-Akt (Ser473and Thr308). However, there are varying degrees of inhibition on the PI3K/Akt/mTOR/p70S6K signaling pathway. Taken together, NCI-H460 was more sensitive to OP-B not only based on inhibition of the pathway but also based on induction of autophagy.

### Correlation between inhibition of the PI3K/Akt/mTOR/p70S6K pathway and induction of autophagy from OP-B in H460 cells

**Figure 5:**
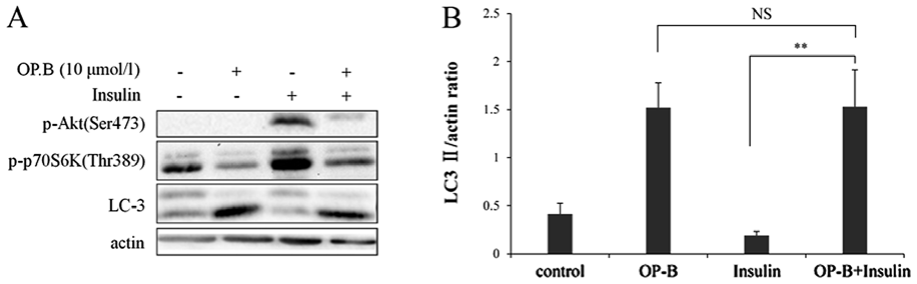
Correlation between inhibition of PI3K/Akt/mTOR/p70S6K and the induction of autophagy in H460 cells. (A) cells treated with 10 μmol/l OP-B for 4 h followed by treatment with or without 200 nM insulin for 30 min were analyzed by immunoblotting for levels of p-Akt (Ser473), p-p70S6K (Thr389), LC3 or actin. (B) Densitometry analysis of LC3-II levels relative to actin in H460 cells was performed using three independent experiments. Error bars, SD; ^**^p<0.01.

Insulin upregulates PI3K and its downstream targets, including Akt and mTOR, and also suppresses autophagy (11–13). As shown in Fig. 5B, 30 min of insulin treatment significantly phosphorylates Akt at Ser473 and p70S6K atThr389. In contrast, when cells were pretreated with OP-B and then stimulated with insulin, the phosphorylation was significantly inhibited. Otherwise, no significant differences in the LC3-II/ actin ratio between OP-B treatment and OP-B treatment with insulin were observed (Fig. 5).

## Discussion

OP-B is a natural active compound extracted from the Chinese herbal medicine ophiopogon. In this study, we found that OP-B successfully inhibited cell proliferation in a panel of NSCLC cell lines. The IC_50_ in all lines tested was <4 μmol/l. In order to further investigate the pharmacological effect of OP-B on NSCLC, we chose NCI-H157 and NCI- H460 cells as our cell line models since they represent the most commonly used NSCLC cells and because they were all sensitive to OP-B in our preliminary studies. Of note, we found that after 24 h of treatment with at least 10 μmol/l OP-B in medium containing 10% FBS, a large number of vacuoles accumulated in the cytoplasm of cells. From the volume of the vacuoles, we judged that NCI-H157 seemed to be more sensitive to OP-B than NCI-H460 (Fig. 2A).

Cell cycle analysis by flow cytometry showed that OP-B induced a modest increase in G0/G1 phase in both cell lines (Fig. 2B). However, expression of caspase-3 and Bcl-2, detected by western blot (Fig. 2C), nuclear morphology (stained by Hoechst 33258), and fluorescence intensity (labeled by AnnexinV/PI) detected by high content screening (HCS) Kinetic Scan Reader (Fig. 2F) all showed that OP-B did not induce apoptosis or necrosis in either cell line.

In order to determine if the vacuoles were associated with autophagy, we measured several markers. Importantly, TEM is able to distinguish autophagic cytoplasmic vacuoles from cellular vesicles, such as endosomes, lysosomes, and apoptotic blebs (14). We first observed cell morphology. The vacuoles had double membranes, the internal contents were degraded by lysosomal hydrolases, and only some myelin figures remained (Fig. 3A). These results are consistent with earlier publications (15). The presence of LC3 in autophagosomes, and the conversion of LC3 I to LC3-II are known indicators of autophagy (16). Detection of LC3 using western blot showed that in both cell lines, the OP-B increased the conversion of LC3 I to II in a dose- and time-dependent manner (Fig. 3B and D). Unexpectedly, the conversion rate of LC3 I to II was more significant in NCI-H460 than in H157 (Fig. 3C and E). To further investigate the reason behind this, we assessed the PI3K/Akt/mTOR signaling pathway, which is the main pathway involved in the regulation of autophagy. Within 4 h of OP-B treatment in NCI-H157 cells, p-Akt was inhibited and autophagy was not induced. However, in H460 cells, p-PDK1, p-Akt, and p-p70S6K were all inhibited by OP-B and autophagy was induced (Fig. 4C and D). In addition, activation of the pathway using Insulin showed that the autophagy induced by OP-B correlated with an active signaling pathway (Fig. 5).

Since activation of PI3K/Akt occurs in 90% of NSCLC cell lines, it has become an important target for the development of anticancer drugs. It is well known that LY294002 and rapamycin are inhibitors of PI3K and mTORC1, respectively. However, these compounds have some drawbacks. For example, LY294002 does not distinguish between class I and class III PI3K, and its inhibition of class III PI3K also inhibits autophagy (17–19). Rapamycin inhibits mTORC1, but has a negative feedback on Akt (20). Herein, we found that at least in NCI-H460 cells, OP-B was an ideal inhibitor of the PI3K/Akt/mTOR/p70S6K pathway. It inhibited all components of the pathway and even had a synergistic effect with LY294002 on Akt. It also decreased the activation of rapamycin on Akt and had a synergistic effect on induction of autophagy.

Taken together, OP-B displayed significant cytotoxicity on a panel of NSCLC cell lines at a relatively low concentration. In NCI H157 and H460 cells, it inhibited p-Akt both at Ser308 and Thr473 and significantly induced autophagy. In NCI-H460 cells, it inhibited the PI3K/Akt/mTOR/p70S6K pathway more thoroughly than in H157 cells. Thus, we speculate that OP-B may be an alternative agent in the classification and treatment of NSCLC.

## References

[1] Kroemer G, Levine B (2008). Autophagic cell death: the story of a misnomer. Nat Rev Mol Cell Biol.

[2] Liang XH, Jackson S, Seaman M (1999). Induction of autophagy and inhibition of tumorigenesis by beclin 1. Nature.

[3] Mizushima N (2007). Autophagy: process and function. Genes Dev.

[4] Baehrecke EH (2005). Autophagy: dual roles in life and death?. Nat Rev Mol Cell Biol.

[5] Levy JM, Thorburn A (2011). Targeting autophagy during cancer therapy to improve clinical outcomes. Pharmacol Ther.

[6] West KA, Brognard J, Clark AS (2003). Rapid Akt activation by nicotine and a tobacco carcinogen modulates the phenotype of normal human airway epithelial cells. J Clin Invest.

[7] Khan N, Afaq F, Khusro FH, Mustafa Adhami V, Suh Y, Mukhtar H (2012). Dual inhibition of phosphatidylinositol 3-kinase/Akt and mammalian target of rapamycin signaling in human nonsmall cell lung cancer cells by a dietary flavonoid fisetin. Int J Cancer.

[8] Jorissen RN, Walker F, Pouliot N, Garrett TP, Ward CW, Burgess AW (2003). Epidermal growth factor receptor: mechanisms of activation and signalling. Exp Cell Res.

[9] Saiki S, Sasazawa Y, Imamichi Y (2011). Caffeine induces apoptosis by enhancement of autophagy via PI3K/Akt/mTOR/p70S6K inhibition. Autophagy.

[10] Codogno P, Meijer AJ (2005). Autophagy and signaling: their role in cell survival and cell death. Cell Death Differ.

[11] Inoki K, Li Y, Xu T, Guan KL (2003). Rheb GTPase is a direct target of TSC2 GAP activity and regulates mTOR signaling. Genes Dev.

[12] Inoki K, Li Y, Zhu T, Wu J, Guan KL (2002). TSC2 is phosphorylated and inhibited by Akt and suppresses mTOR signalling. Nat Cell Biol.

[13] Garami A, Zwartkruis FJ, Nobukuni T (2003). Insulin activation of Rheb, a mediator of mTOR/S6K/4E-BP signaling, is inhibited by TSC1 and 2. Mol Cell.

[14] Clarke PG (1990). Developmental cell death: morphological diversity and multiple mechanisms. Anat Embryol.

[15] Fujiwara K, Iwado E, Mills GB, Sawaya R, Kondo S, Kondo Y (2007). Akt inhibitor shows anticancer and radiosensitizing effects in malignant glioma cells by inducing autophagy. Int J Oncol.

[16] Kabeya Y, Mizushima N, Ueno T (2000). LC3, a mammalian homologue of yeast Apg8p, is localized in autophagosome membranes after processing. EMBO J.

[17] Blommaart EF, Krause U, Schellens JP, Vreeling-Sindelarova H, Meijer AJ (1997). The phosphatidylinositol 3-kinase inhibitors wortmannin and LY294002 inhibit autophagy in isolated rat hepatocytes. Eur J Biochem.

[18] Petiot A, Ogier-Denis E, Blommaart EF, Meijer AJ, Codogno P (2000). Distinct classes of phosphatidylinositol 3′-kinases are involved in signaling pathways that control macroautophagy in HT-29 cells. J Biol Chem.

[19] Arico S, Petiot A, Bauvy C (2001). The tumor suppressor PTEN positively regulates macroautophagy by inhibiting the phosphatidylinositol 3-kinase/protein kinase B pathway. J Biol Chem.

[20] O’Reilly KE, Rojo F, She QB (2006). mTOR inhibition induces upstream receptor tyrosine kinase signaling and activates Akt. Cancer Res.

